# Major depression, fibromyalgia and labour force participation: A population-based cross-sectional study

**DOI:** 10.1186/1471-2474-7-4

**Published:** 2006-01-19

**Authors:** Aliya Kassam, Scott B Patten

**Affiliations:** 1 Section of Community Psychiatry, Health Services Research Department, Institute of Psychiatry, King's College London, UK; 2 Department of Community Health Sciences, University of Calgary, 3330 Hospital Drive NW, T2N 4N1, Calgary, AB, Canada; 3 Department of Psychiatry, University of Calgary, 1403 – 29 Street NW. Calgary, T2N 2T9, Canada

## Abstract

**Background:**

Previous studies have documented an elevated frequency of depressive symptoms and disorders in fibromyalgia, but have not examined the association between this comorbidity and occupational status. The purpose of this study was to describe these epidemiological associations using a national probability sample.

**Methods:**

Data from iteration 1.1 of the Canadian Community Health Survey (CCHS) were used. The CCHS 1.1 was a large-scale national general health survey. The prevalence of major depression in subjects reporting that they had been diagnosed with fibromyalgia by a health professional was estimated, and then stratified by demographic variables. Logistic regression models predicting labour force participation were also examined.

**Results:**

The annual prevalence of major depression was three times higher in subjects with fibromyalgia: 22.2% (95% CI 19.4 – 24.9), than in those without this condition: 7.2% (95% CI 7.0 – 7.4). The association persisted despite stratification for demographic variables. Logistic regression models predicting labour force participation indicated that both conditions had an independent (negative) effect on labour force participation.

**Conclusion:**

Fibromyalgia and major depression commonly co-occur and may be related to each other at a pathophysiological level. However, each syndrome is independently and negatively associated with labour force participation. A strength of this study is that it was conducted in a large probability sample from the general population. The main limitations are its cross-sectional nature, and its reliance on self-reported diagnoses of fibromyalgia.

## Background

Fibromyalgia is a syndrome of unknown etiology characterized by chronic widespread joint and muscle pain and by pain on palpation of tender points [1]. The literature contains several population-based prevalence estimates, which have generally fallen in the range of 1–3% [2-5]. Chronic medical conditions are, in general, associated with an increased frequency of major depression [6-8], but the association with fibromyalgia may be particularly strong. One general population study reported an odds ratio of 2.85 for subjects with fibromyalgia equalling or exceeding a score of 4.0 on the depression scale of the Arthritis Impact Measurement Scales [2]. Another study, using the Present State Examination to detect ICD-10 defined depressive disorders in a general population sample, reported a doubling of prevalence in subjects with chronic widespread pain [9].

Some studies have focussed on co-aggregation of these conditions in families [10], whereas other studies have examined possible pathophysiological linkages between depression and fibromyalgia. It has been reported, for example, that people with fibromyalgia have elevated levels of alexithymia and anger [11], that depression risk factors such as work stress [12] and childhood traumatic events [13] may contribute to fibromyalgia etiology, and that antidepressants are efficacious treatments for fibromyalgia [14]. Other studies have identified an influence of social support and emotional context on pain thresholds in fibromyalgia [15]. These results, and others, have caused some authors to conclude that there is an intrinsic connection, or perhaps on some level an equivalency, between depressive disorders and fibromyalgia [10,16-18]. Other authors have conceptualized fibromyalgia and depressive disorders as members of a broad category of stress disorders [19].

Based on an analysis of case-control data, it has been argued that the comorbidity between mood disorders and fibromyalgia is an artefact of bias resulting from treatment-seeking behaviour [20]. This assertion highlights the importance of population-based data and the need to further characterise the epidemiology of this comorbidity in the general population. Epidemiological data are of considerable importance for describing and understanding public health problems. A study conducted in the United States using administrative health care and disability claims data compared the economic burden associated with co-morbid depression and fibromyalgia. It was found that mean employer payments per patient were $11,899, $8,073 and $5,163 for employees with co-morbid depression and fibromyalgia, depression alone, and fibromyalgia alone, compared to $2,486 for the total sample [21]. These results suggest that the two conditions may have effects that are independent in the multiplicative sense: the two conditions alone increased costs by a factor of 2–3, and in combination by an amount resembling the product of these increases.

One objective of this study was to describe the association between co-morbid depression and fibromyalgia in the general population in more detail than has been done previously. Another objective was to examine major depression-fibromyalgia comorbidity in relation to a key functional outcome: labour force participation.

## Methods

The data source for this study was the Canadian Community Health Survey (CCHS), iteration 1.1, a cross-sectional general health survey of household residents conducted by Statistics Canada (the Canadian Government's national statistical agency) in 2000 and 2001. The sample size for the CCHS 1.1 was 131,535 subjects aged 12 years and older. However, as the instrument used to identify depressive episodes has not been validated in adolescents, we restricted the current analysis to subjects 18 years and older, n = 115,160.

The Composite International Diagnostic Interview (CIDI) short form for major depression (CIDI-SFMD) [8] was used to identify episodes of major depression in this survey. The CIDI-SFMD is a brief version of the CIDI diagnostic interview. Five or more of the DSM-IV-TR symptom-based criteria for major depression (at least one of which must be depressed mood or loss of interest) were required. According to the CIDI-SFMD validation data, this would result in a 90% positive predictive value for DSM-IV major depression [9].

The CCHS 1.1 also collected self-report diagnostic information about chronic medical conditions. In each case, survey items enquired about the presence of long-term medical conditions that had been "diagnosed by a health professional." One of the conditions evaluated was fibromyalgia. The CCHS 1.1 also included items evaluating labour force participation. As subjects over the age of 75 are unlikely to be members of the labour force, analyses of this variable excluded subjects over the age of 75. The sample size for this part of the analysis was 105,538.

Household income was dichotomized after adjustment for the number of people in each [22]. Lowest and lower-middle income categories were collapsed into a "low income" group and upper-middle and highest income quartiles were grouped into a "high income" group. Education was dichotomized into a "low education" group that included high school graduation or less and a "high education" group containing those with at least some post secondary education. Marital status was classified using two categories: those who were married/common-law were placed into a "married" category and those who were single (never married), widowed, separated and divorced comprising the "unmarried" group.

Analysis was conducted using SAS^® ^version 8.0. Due to multi-staged sampling procedures, unequal selection probabilities and non-response, sampling weights adjusting for these factors were used in the analysis. A bootstrap variance estimation procedure was used for statistical analysis of the weighted estimates.

## Results

The CCHS achieved a 91.4% household response rate, and a 91.9% individual-respondent response rate. This resulted in a survey sample that was highly representative of the national target population, even before the application of sampling weights (see Table 1). Table 1 presents a description of the sample, and reproduces expected patterns of association. Fibromyalgia is significantly more common in women and in older age groups, whereas major depression is more common in women, in the younger age category and in unmarried subjects. Both major depression and fibromyalgia were more common in low income subjects.

The association between fibromyalgia and major depression may have been confounded by other variables that are associated with major depression. In order to explore this possibility, the prevalence of major depression in subjects with and without fibromyalgia was stratified by a set of demographic variables. As seen in Table 2, the prevalence of major depression was found to be consistently higher in those with fibromyalgia than in those without, irrespective of demographic category.

Non-participation in the labour force was reported by 40,630 subjects (weighted frequency estimate 35.0%). A logistic regression model incorporating both major depression and fibromyalgia as predictors of labour force participation identified no interaction between these two variables (Wald Statistic = 0.17, p = 0.68) suggesting an independent multiplicative contribution to the outcome, so the interaction term was removed from the model. Odds ratios were then estimated from the reduced model both for fibromyalgia (OR = 2.7, 95% CI 2.3 – 3.2) and major depression (OR = 1.4, 95% CI 1.3 – 1.5).

In a series of additional analyses, the logistic regression model described above was expanded to include each of the demographic variables listed in Table 1, along with associated interaction terms. The associations between major depression and fibromyalgia remained significant in all of these models, and in no cases did major depression by fibromyalgia interactions emerge.

Finally, the subset of respondents who reported that they were permanently disabled and unable to work was identified. The weighted estimate for this proportion was 2.6% (95% CI 2.5 – 2.8) in the general population (2.2% in those without major depression or fibromyalgia). Among subjects with major depression but not fibromyalgia, 5.7% (95% CI 4.9 – 6.5) fell into this category, as did 15.9% (95% CI 12.2 – 19.6) of subjects with fibromyalgia but not major depression. In subjects with both conditions, 23.0% (95% CI 15.2 – 30.9) fell into this category. Again a multiplicative pattern indicative of an independent effect is observed in the sense that an approximately 2-fold and 5-fold increase in subjects with one of these conditions combines in the comorbid group to an approximately 10 fold increase over the baseline frequency.

A similar pattern was found when data from a more general item referring to restriction of activities (at work, at home or in other categories) due to a physical or mental health problems was examined. Subjects without major depression or fibromyalgia reported activity restrictions 10.0% of the time (95% CI 9.7 – 10.2), having major depression alone increased this to 20.0% (95% CI 18.7 – 21.5), fibromyalgia alone to 28.4% (95% CI 32.3 – 41.4) and both conditions together to 57.6% (95% CI 48.5 – 66.8).

## Discussion

A strong association between fibromyalgia and major depression was observed in this study, and it remained evident after stratification for sex, age, marital status, education and income. This finding replicates and solidifies earlier results. One of the previous studies used a depressive symptom measure, rather than a diagnostic instrument [2] and another used ICD-10 criteria for depression and the concept of chronic widespread pain was used rather than a fibromyalgia diagnosis [9]. It is worth noting that chronic pain in general is associated with depression, and that pain and depression may be linked through a variety of biological mechanisms, see review [23]. From the perspective of labour force participation, subjects with either or both of these conditions are less likely to be participating in the workforce. They appear to have an independent effect on the probability of workforce participation.

While the large sample size of the CCHS is advantageous for statistical analysis, the use of such data is subject to limitations. As a general health survey, the CCHS uses a variety of brief measures. Both the major depression measure (CIDI-SFMD) and the self-report of fibromyalgia may be subject to error. For this reason, the findings should be replicated in studies using more detailed measures. Because the data source for this study was a general health survey, the capacity to evaluate the impact of confounding variables was somewhat limited, although the extent of stratified analysis possible exceeded that of earlier general population studies because of the large sample size.

In this analysis, the association between major depression, fibromyalgia and labour force participation was examined using three different perspectives: a traditional definition of labour force participation, not working due to illness or disability and, most broadly, activity limitations. The results were broadly consistent across the various definitions, suggesting that both conditions impair functioning across a broad spectrum of occupational activities. An interesting finding was that the strength of association was stronger for fibromyalgia than for major depression. This result emphasizes the potential importance of fibromyalgia as a contributor to impairment in occupational functioning.

## Conclusion

From the public health perspective, these data demonstrate that major depression and fibromyalgia frequently co-occur and, when they do, both syndromes appear to contribute to a reduced frequency of labour force participation. From the perspective of service planning, these results suggest that the availability of services addressing both problems may lead to better occupational and functional outcomes. Potentially, existing services can be more effective if they are integrated in a way that fosters the delivery of such care. Integration of cognitive-behavioural strategies for pain (see review, [24]) with those for depression, for example, may lead to intervention strategies that are useful a very high proportion of patients.

## List of Abbreviations

CCHS Canadian Community Health Survey

CIDI Composite International Diagnostic Interview

CIDI-SFMD Composite International Diagnostic Interview Short Form for Major Depression

ICD-10 International Classification of Disease, 10^th ^Edition

## Competing interests

Neither author has competing interest to declare. The analyses reported here are based on data collected by Statistics Canada, the Canadian Government's statistical agency. The analysis itself does not reflect the opinions of Statistics Canada.

## Authors' contributions

Both AK and SP participated in conceptualization of the project, and in preparation of the research proposal. Both authors worked together in carrying out the analyses, interpretation of the results, and in preparation of the manuscript.

## Disclaimer

The analyses reported here are based on data collected by Statistics Canada, the Canadian Government's statistical agency. The analysis itself does not reflect the opinions of Statistics Canada.

## Pre-publication history

The pre-publication history for this paper can be accessed here:



## Figures and Tables

**Table 1:** Demographic & Clinical Features of the Sample.

		Sample Characteristics		Prevalence (95% CI)	
		
		Unweighted n (%)	Weighted %	Fibromyalgia %	Major Depression %
Sex	Men	52772 (45.8)	49.1	0.4 (0.3–0.4)	5.3 (5.0–5.6)
	Women	62388 (54.2)	50.9	2.0 (1.9–2.1)	9.4 (9.1–9.7)
Age	50 years +	48707 (42.3)	36.5	1.7 (1.5–1.9)	4.9 (4.6–5.2)
	18 to 49	66430 (57.7)	63.5	0.3 (0.2–0.3)	8.8 (8.5–9.1)
Income*	High	85851 (80.6)	86.8	1.0 (0.9–1.1)	6.5 (6.2–6.7)
	Low	20626 (19.4)	13.2	1.5 (1.3–1.7)	9.7 (9.3–10.1)
Education**	High	60986 (53.5)	57.1	1.2 (1.0–1.3)	7.2 (6.9–7.4)
	Low	53008 (46.5)	42.9	1.3 (1.1–1.4)	7.7 (7.4–8.0)
Marital Status***	Married	66888 (58.2)	64.2	1.2 (1.1–1.4)	5.6 (5.3–5.8)
	Unmarried	48113 (41.8)	35.8	1.1 (1.0–1.3)	10.7 (10.3–11.1)
Total		115137 (100)	100	1.2 (1.1 – 1.3)	7.4 (7.2 – 7.6)

* n = 106477 because of missing income data from 7.5% of subjects.

** n = 113994 because of missing data from 1% of subjects.

*** n = 115001 because of missing data from 0.1% of subjects.

**Table 2:** Annual Major Depression Prevalence With and Without Fibromyalgia, Stratified by Demographic Variables

		Prevalence in Subjects with Fibromyalgia % (95% CI)	Prevalence in Subjects without Fibromyalgia % (95% CI)
Sex	Men	13.7 (8.3 – 19.1)	5.3 (5.0 – 5.6)
	Women	23.6 (20.6 – 26.7)	9.1 (8.8 – 9.4)
Age	50 years +	15.0 (11.9 – 18.1)	4.9 (4.6 – 5.2)
	18 to 49	29.9 (25.4 – 34.5)	8.8 (8.5 – 9.1)
Income	High	20.8 (16.8 – 24.8)	6.5 (6.2 – 6.7)
	Low	27.1 (22.7 – 31.4)	9.7 (9.3 – 10.1)
Education	High	24.2 (20.2 – 28.2)	7.2 (6.9 – 7.4)
	Low	19.7 (25.7 – 23.8)	7.7 (7.4 – 8.0)
Marital Status	Married	17.7 (14.5 – 20.9)	5.6 (5.3 – 5.8)
	Unmarried	30.7 (25.8 – 35.7)	10.7 (10.3 – 11.1)
